# Microfluidic high-throughput selection of microalgal strains with superior photosynthetic productivity using competitive phototaxis

**DOI:** 10.1038/srep21155

**Published:** 2016-02-08

**Authors:** Jaoon Young Hwan Kim, Ho Seok Kwak, Young Joon Sung, Hong Il Choi, Min Eui Hong, Hyun Seok Lim, Jae-Hyeok Lee, Sang Yup Lee, Sang Jun Sim

**Affiliations:** 1 Department of Chemical and Biological Engineering, Korea University, Seoul, 136-713, Republic of Korea; 2 Department of Botany, University of British Columbia, Vancouver, V6T1Z4, Canada; 3 Department of Chemical and Biomolecular Engineering (BK21 Plus Program), BioProcess Engineering Research Center, Bioinformatics Research Center, Center for Systems and Synthetic Biotechnology, Institute for the BioCentury, Korea Advanced Institute of Science and Technology, Daejeon 305-701, Republic of Korea; 4 Green School, Korea University, Seoul, 136-713, Republic of Korea

## Abstract

Microalgae possess great potential as a source of sustainable energy, but the intrinsic inefficiency of photosynthesis is a major challenge to realize this potential. Photosynthetic organisms evolved phototaxis to find optimal light condition for photosynthesis. Here we report a microfluidic screening using competitive phototaxis of the model alga, *Chlamydomonas reinhardtii*, for rapid isolation of strains with improved photosynthetic efficiencies. We demonstrated strong relationship between phototaxis and photosynthetic efficiency by quantitative analysis of phototactic response at the single-cell level using a microfluidic system. Based on this positive relationship, we enriched the strains with improved photosynthetic efficiency by isolating cells showing fast phototactic responses from a mixture of 10,000 mutants, thereby greatly improving selection efficiency over 8 fold. Among 147 strains isolated after screening, 94.6% showed improved photoautotrophic growth over the parental strain. Two mutants showed much improved performances with up to 1.9- and 8.1-fold increases in photoautotrophic cell growth and lipid production, respectively, a substantial improvement over previous approaches. We identified candidate genes that might be responsible for fast phototactic response and improved photosynthesis, which can be useful target for further strain engineering. Our approach provides a powerful screening tool for rapid improvement of microalgal strains to enhance photosynthetic productivity.

The heavy reliance on fossil fuels to meet soaring global energy demands threatens sustainability and energy security^1^. Solar energy, the ultimate energy source on Earth, can supply more energy than annual global energy consumption in 1 hour^2^. Thus, efficient conversion of solar energy into available fuels could offer the most sustainable solution to this issue. Microalgae account for nearly half of primary production through photosynthesis^3^. They have attracted considerable attention as a potential source of renewable energy due to the ability to accumulate non-polar lipids that can be converted to biodiesel and numerous advantages over plants^1^. However, vast improvement in solar energy conversion efficiency is still necessary for the realization of microalgal biofuels because photosynthetic productivity is limited by the inherently low efficiency of solar energy conversion process^1^,^4^. The theoretical maximum photosynthetic efficiency (8–10%) is attainable only in low light, where most absorbed photons can be utilized. Under natural light conditions, it is difficult to obtain such a high efficiency due to light intensities exceeding the photosynthetic capacity and fluctuations in light intensity^5^,^6^.

Previous approaches to increase photosynthetic efficiency by engineering microalgal strains have relied on random mutagenesis screens due to the lack of efficient genetic engineering tools and difficulties in engineering of photosynthetic process^1^,^7^. Several targeted genetic approaches for down-regulation of light-harvesting antenna complex by RNA silencing or expression of translational repressor have shown some success in increasing photosynthetic productivity^8^,^9^. However, oxygenic photosynthesis is the most complex energy conversion process, in which over 100 genes are involved^10^, thus making knowledge-based targeted genetic approaches less feasible. For traditional random mutagenesis screens, it is required to transfer each colony into separate wells in microplates for characterization of individual strains, which is unsuitable for the improvement of strain by screening large mutant libraries containing more than 10^4^ mutants due to the laborious, time-consuming and expensive nature. Therefore, more efficient screening strategy is desperately needed for rapid and efficient isolation of strains with high photosynthetic productivity.

Photosynthetic organisms have evolved various means for regulation of exposure to light^11^, which is the most important environmental stimulus. The unicellular photosynthetic eukaryotes evolved phototaxis for maintaining optimal light exposure for photosynthesis^12^. They evolved such phototactic ability multiple times independently using the same general strategy^13^, suggesting that phototaxis confers evolutionary fitness advantage. It has been reported that the sign of phototaxis in *C. reinhardtii* is linked with photosynthetic activity and controlled by cytoplasmic redox balance that is affected by photosynthetic activity to maintain moderately reduced state of the cytoplasm^12^. Therefore, we have reasoned that the efficiency of photosynthetic electron transport and photosynthetic productivity are correlated with phototactic response. Because negative phototactic response is triggered under reduced cytoplasmic condition that can be caused by increase in photosynthetic activity^12^, fine tuning of light capture to obtain optimal cytoplasmic state and photosynthesis can be achieved by fast phototactic response, resulting in improved photosynthetic efficiency and productivity (Fig. 1a).

Numerous microfluidic approaches have been performed for high-throughput isolation, analysis, cultivation and transformation of microalgal cells^14^,^15^,^16^,^17^,^18^. However, there was no report on the successful application of microfluidic screening method to isolation of microalgal strains with highly improved photosynthetic efficiency and productivity from large mutant libraries, which is essential to achieve economically viable algal biofuels. Here we describe novel microfluidic approaches for investigating the positive relationship between phototaxis and photosynthetic efficiency by precise analysis of phototactic response of *C. reinhardtii*, an attractive model microalga for the study of phototaxis and photosynthesis^12^,^19^, and high-throughput screening using phototaxis as the readout for rapid isolation of strains with high photosynthetic efficiency. It is worth noting that this screening strategy based on competition in phototactic response has been made possible for the first time using a microfluidic screening device, which enables isolating cells showing fast phototactic response by simple manipulation, a feat difficult to achieve using bulk system. Furthermore, this method can be directly applied to mutant mixture without characterization of individual strains, thus large mutant libraries (≥10^4^ mutants) can be screened regardless of library size in considerably less time compared with traditional techniques. We could accomplish 5 cycles of phototaxis-assisted screening of 10,000 mutants within a single day. Among 147 strains isolated after screening, 94.6% showed improved photoautotrophic growth over the parental strain. Finally selected mutant showed a vast improvement in both biomass production and lipid production under nitrogen starvation up to 2.5- and 8-fold, respectively, substantial improvement over previous approaches^9^,^20^,^21^,^22^. This approach provides a powerful screening strategy with immediate and broad applications toward microalgal strain development for improving photosynthetic production of biomass, chemicals, and biofuels.

## Results

### Microfluidic approach for analysis of phototactic response

**Figure 1:**
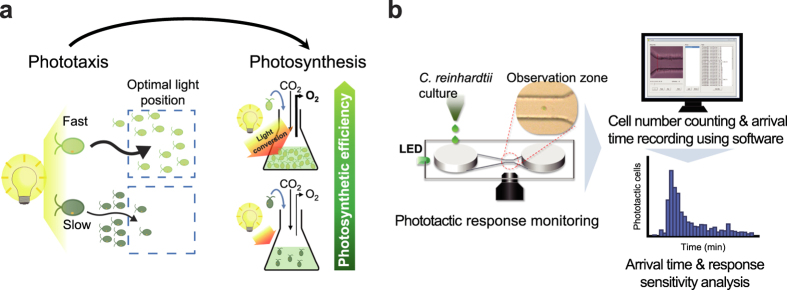
Schematic description of hypothesis and microfluidic approach for analysis of phototactic response. (**a**) Schematic showing contribution of fast phototactic response to improving photosynthetic efficiency. Fine tuning of light capture by fast phototactic response (light green cell) can provide optimal condition for photosynthesis, whereas cells exhibiting slow responses (dark green cell) are less competitive in finding optimal light condition, resulting in low photosynthetic productivity. (**b**) Experimental scheme showing analysis of negative phototactic response using a microfluidic device. Cells grown exponentially were loaded into inlet chamber in a microfluidic device and exposed to green LED (70 μmol photons m^-2 ^s^-1^). The phototactic movements of cells were monitored under an inverted microscope and analysed using video analysing software.

To confirm the relationship between phototaxis and photosynthetic efficiency, it is necessary to precisely analyse the phototactic response. In previous approaches, the measurement of change in cell density^23^ or tracking movement of freely moving cells in bulk phase^12^ was used for the analysis of phototactic response, which makes it difficult to obtain accurate and reliable results. In contrast, microfluidic device can minimize the influence of external environment and provide well-defined microstructures on the cellular length scale^24^, which are suitable for monitoring cellular response at the single-cell and population level in response to various stimuli. We developed a method using a poly(dimethylsiloxane) (PDMS) microfluidic device with a long and gradually narrowed microchannel including observation zone (Fig. 1b) to reduce the hindrance of phototactic movement by microchannel and facilitate the monitoring of phototactic response at the single-cell resolution level.

In this study, we used *C. reinhardtii* CC-125 as a reference strain, which is regarded as the wild type 137c genotype and is employed in many mutational screens in this species^25^. Using the microfluidic device, we could monitor the immediate phototactic responses of a single cell of *C. reinhardtii* in the microchannel to the directional light (green LED, 70 μmol photons m^-2 ^s^-1^) (Supplementary Video S1) and analysed the arrival time of individual cells from the inlet to the observation zone near the outlet in the microchannel in response to various conditions (Fig. 1b and Supplementary Video S2). We thereby achieved a quantifiable measure of phototactic response of individual strains at the population level, which are difficult to obtain using bulk-phase methods as previously reported^12^,^23^. To assess the rate of negative phototaxis, skewness of arrival time distribution and inverse average arrival time, were calculated from the histogram of phototactic cells as a function of their arrival time. The arrival time of each cell is a function of starting time of phototactic response and phototactic velocity in the linear microchannel exposed to a directional light. Therefore, average arrival time of a population of cells indicates how fast they respond and move by exposure to the light stimuli. The skewness of arrival time distribution of a population of cells indicates the proportion of cells showing fast phototactic response.

### Analysis of phototaxis and photosystem II efficiency under different conditions

We first examined whether the rate of negative phototaxis of the wild-type *C. reinhardtii* strain (CC-125) was in accord with the necessity of photosynthetic activity under circadian rhythm and trophic conditions. Both conditions are known as affecting the photosynthetic activity^26^,^27^ and phototactic behaviour^28^, but exact covariance has not previously been reported. The distribution of the number of phototactic cells according to arrival time varied with culture time under light/dark (LD) cycle. After lights-on, the skewness of arrival time distribution increased until 6 h, followed by decrease to the initial level observed at lights-on at the end of light phase. After lights-off, the skewness of arrival time distribution gradually decreased, and almost no response was detected at the end of dark phase (Supplementary Fig. S1a and Supplementary Video S3). We measured the photosystem II (PSII) operating efficiency (Y(II)) according to culture time under LD cycle (Supplementary Fig. S1b), which is a useful parameter indicating the proportion of the light absorbed by chlorophyll associated with PSII that is used in photochemistry and can be an indicator of linear electron transport rate and overall photosynthesis^29^. We found that Y(II) showed a similar pattern to the inverse average arrival time and the skewness of arrival time distribution of phototactic cells (Supplementary Fig. S1c). The phototactic response under photoautotrophic condition was faster than photomixotrophic condition, which agrees with the photosynthetic efficiency showing higher Y(II) values under photoautotrophic condition than photomixotrophic condition (Supplementary Fig. S1 d–f). These results indicate that the rate of phototaxis and photosynthetic activity under controlled laboratory conditions vary with light condition and the availability of exogenous organic carbon.

### Analysis of phototaxis and PSII efficiency in diverse strains

**Figure 2:**
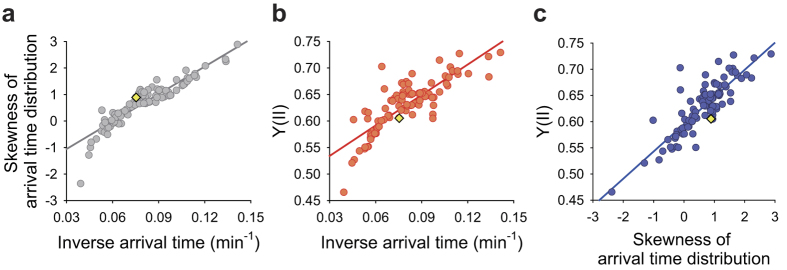
Correlation between phototaxis and photosynthetic efficiency in diverse strains. (**a**) Scatter plot showing the correlation between skewness of arrival time distribution and inverse average arrival time of 100 strains, including the wild-type strain (CC-125) and 99 randomly selected insertional mutants with wide range of PSII operating efficiency (Y(II)) (R^2^ = 0.84). (**b**) Scatter plot showing the correlation between Y(II) and inverse average arrival time of the 100 strains (R^2^ = 0.69). (**c**) Scatter plot showing correlation between Y(II) and skewness of arrival time distribution of the 100 strains (R^2^ = 0.72). Cells were grown photomixotrophically under continuous low light condition (50 μmol photons m^-2 ^s^-1^). Phototactic response was measured on 6,600 cells per analysis. The yellow diamond: the wild type (**a**–**c**). Data are the mean of three biological replicates.

We next collected a set of 100 strains with a wide range of Y(II) from random insertional mutants generated from the wild-type strain (CC-125) and analysed their negative phototactic responses (Supplementary Fig. S2 and Supplementary Video S4). The skewness of arrival time distribution in negative phototaxis was found to be strongly correlated with the inverse average arrival time (Fig. 2a). Also, these two parameters related to the rate of negative phototaxis were correlated with Y(II) in these strains (Fig. 2b,c). These results indicate that a fast phototactic response contributes to an increase in photosynthetic efficiency. We also found that various strains with different rate of phototaxis showed no response without exposure to light stimulus (Supplementary Fig. S3), confirming that the negative phototactic response measured by exposure to light stimulus in the microfluidic device is irrelevant to random motility.

### Competitive phototaxis-assisted high-throughput screening

**Figure 3:**
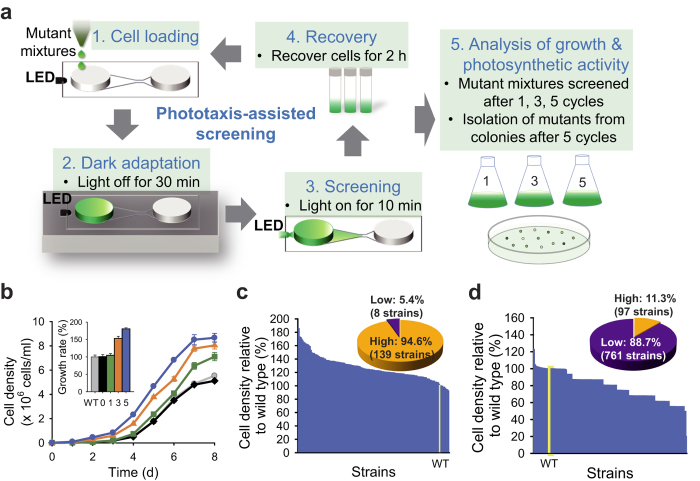
Phototaxis-assisted screening. (**a**) Schematic of phototaxis-assisted screening for the isolation of mutants showing fast phototactic response. (**b**) Comparison of growth characteristics of the wild type (WT(CC-125), grey), the mutant mixture without application of phototaxis-assisted screening (0, black) and the mutant mixtures obtained after 1 (green), 3 (orange) and 5 (blue) cycles of phototaxis-assisted screening (inset: maximum growth rates relative to wild type (%)). Screening was performed on a mixture of 10,000 insertional mutants generated from the wild-type strain using a microfluidic device. To compare the growth of mutant mixtures between each cycle of phototaxis-assisted screening, cells were inoculated to a density of ~1 × 10^4 ^cells/ml and grown photoautotrophically. Growth curves were fit to the Gompertz function^40^ to calculate growth rates. (**c**) Photoautotrophic cell growth of 147 randomly selected mutants after 5 cycles of phototaxis-assisted screening relative to the wild-type strain (WT, yellow bar). (**d**) Photoautotrophic cell growth of 858 randomly selected mutants without application of phototaxis-assisted screening relative to the wild-type strain (WT, yellow bar). The pie charts show the proportion of mutants presenting higher and lower growth compared to the wild-type strain (**c**,**d**). Cells were grown in continuous low light (50 μmol photons m^-2 ^s^-1^). Data and error bars are mean ± SD of three biological replicates.

Having demonstrated the strong relationship between the rate of negative phototaxis and photosynthetic efficiency, we designed a high-throughput screening method based on competitive phototaxis using a microfluidic device for rapid isolation of mutants with high photosynthetic efficiencies (Fig. 3a and Supplementary Fig. S4a). We constructed a library of 10,000 random insertional mutants of *C. reinhardtii* CC-125 strain by electroporation (see Methods). After collecting cultures of all mutants, we cycled the screening process up to 5 times for the enrichment of strains showing fast phototactic response from a mixture of 10^4^ mutants. As the number of screening cycles increased, the number of cells obtained after each cycle of screening decreased, and cells exhibiting fast phototactic responses could be isolated (Supplementary Fig. S4b). The maximum cell density and maximum growth rate of the mutant mixture isolated after each cycle were improved with an increasing number of screening cycles (Fig. 3b and Supplementary Fig. S4c), indicating that mutants isolated by phototaxis-assisted screening indeed possess higher photosynthetic productivity compared to the parent strain (CC-125). The cell number of strains that arrived at the observation zone within 10 min showed positive relationships with the skewness of arrival time distribution and inverse arrival time, supporting that the population of strains showing fast phototactic responses increases through phototaxis-assisted screening (Supplementary Fig. S5 a,b). The population and growth dynamics of mutant mixture obtained from simple mathematical models also showed similar patterns to our results (Supplementary Fig. S5 c,d).

We accomplished five successive cycles of phototaxis-assisted screening within a single day. We obtained the mixture of mutants showing fast phototactic response after five screening cycles. The mixture was plated on agar plate to isolate each strain as separated colonies. We next picked 147 strains from agar plates and cultured to analyse their photoautotrophic growth. Among 147 randomly isolated strains from the mutant mixture obtained after five screening cycles, 139 strains (94.6%) showed enhanced growth compared with the wild-type strain (CC-125) under photoautotrophic condition (Fig. 3c). On the other hand, only 97 strains (11.3%) among 858 randomly chosen mutants without application of phototaxis-assisted screening showed improved growth compared to the wild-type strain (Fig. 3d), indicating that phototaxis-assisted screening strategy increase the probability of selecting strains with improved photosynthetic efficiency by rapid enrichment of strains showing fast phototactic responses.

### Analysis of mutants isolated by phototaxis-assisted screening

**Figure 4:**
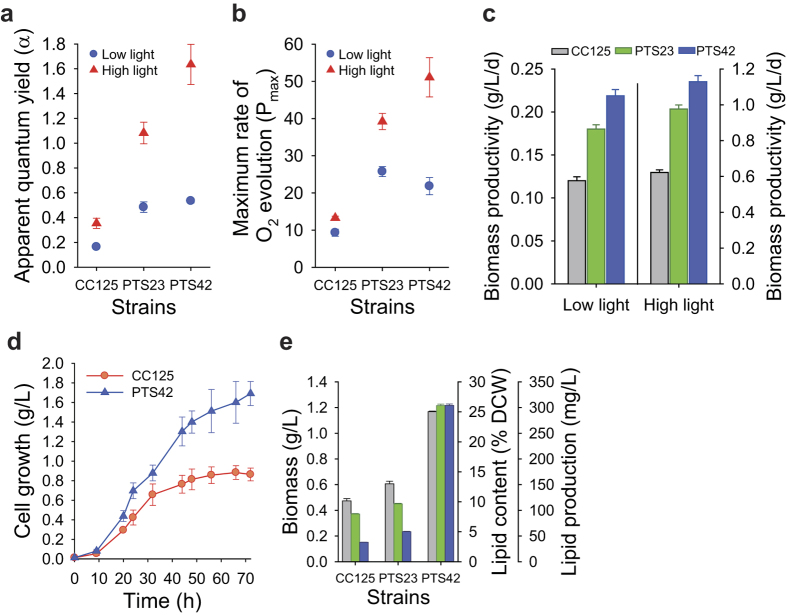
Photosynthetic performances of two mutants (PTS23 and PTS42) isolated after 5 cycles of phototaxis-assisted screening. (**a**) Apparent quantum yield of oxygen evolution on a per-chlorophyll basis (*α*, arbitrary unit) in the two mutants compared to the wild-type strain under continuous low light (50 μmol photons m^-2 ^s^-1^) and high light conditions (300 μmol photons m^-2 ^s^-1^). (**b**) Maximum photosynthetic rates on a per-chlorophyll basis (P_max_, μmol O_2_ (mg Chl)^-1 ^min^-1^) in the two mutants compared to the wild-type strain under continuous low light (50 μmol photons m^-2 ^s^-1^) and high light conditions (300 μmol photons m^-2 ^s^-1^). (**c**) Biomass productivity of the two mutants compared to the wild-type strain under continuous low light (50 μmol photons m^-2 ^s^-1^, left) and high light condition (300 μmol photons m^-2 ^s^-1^, right). Cells were grown photoautotrophically in flasks with the same initial cell densities (~5 × 10^5 ^cells/ml) in a CO_2_ incubator at 5% CO_2_ (**a**–**c**). (**d**) Cell growth (g dry cell weight per L culture) of PTS42 mutant in mass culture using photobioreactor compared to the wild-type strain. Cells were grown photoautotrophically with the same initial cell densities (~1 × 10^5 ^cells/ml) in 3-liter TP medium using a 5-liter photobioreactor at a light intensity of 350 μmol photons m^-2 ^s^-1^ by supplying 5% CO_2_-enriched air at a flow rate of 50 ml liter^-1 ^min^-1^. (**e**) Biomass production (grey bar), lipid contents (total FAME as a % of dry cell weight, green bar), lipid production (mass of total FAME per L culture, blue bar) of two mutants compared to the wild-type strain under nitrogen-starved condition. Cells grown photoautotrophically in flasks for 4 days in a CO_2_ incubator at 5% CO_2_ under continuous low light condition (50 μmol photons m^-2 ^s^-1^) were incubated in TP(-N) media at 5% CO_2_ for 4 days for lipid accumulation. Data and error bars are mean ± SD of three biological replicates.

Two mutants (PTS23 and PTS42) showing the highest growth (ΔOD_800_) among 147 strains were selected for further evaluation of their performances. These mutants showed fast phototactic responses and higher Y(II) compared with the wild-type strain (CC-125) (Supplementary Fig. S6 a–c). They exhibited substantial improvements in photosynthetic oxygen evolution (both on a per-chlorophyll and per-cell basis) (Fig. 4a,b and Supplementary Fig. S6 d,e) and cell growth (Fig. 4c and Supplementary Fig. S6 f–h) compared to the wild-type strain under continuous low (50 μmol photons m^-2 ^s^-1^) and high light conditions (300 μmol photons m^-2 ^s^-1^). Photosynthetic oxygen evolution was measured as apparent quantum yield of oxygen evolution (*α*, the slope of linear portion of oxygen evolution curve against light intensity) and maximum photosynthetic rate (net oxygen evolution plus dark respiration). We observed that these mutants showed improved maximum photosynthetic rates on a per-cell basis compared to the wild type (PTS23: 61% (low light) and 40% (high light) increases; PTS42: 41% (low light) and 57% (high light) increases) (Supplementary Fig. S6d). Improved maximum photosynthetic rate was contributed by increases in net oxygen evolution (on a per-cell basis, PTS23: 57% (low light) and 40% (high light) increases; PTS42: 49% (low light) and 60% (high light) increases) as well as dark respiration (on a per-cell basis, PTS23: 71% (low light) and 39% (high light) increases; PTS42: 21% (low light) and 49% (high light) increases) compared to wild type. Notably, apparent quantum yield of oxygen evolution on a per-chlorophyll basis in PTS42 strain showed 227% and 362% increases compared to the wild type under low and high light conditions, respectively (Fig. 4a). Apparent quantum yield of oxygen evolution on a per-cell basis in PTS42 also showed 97% (low light) and 89% (high light) increases compared to the wild type (Supplementary Fig. S6e). PTS42 also exhibited 82 ± 6.0, 115 ± 18.5 and 62 ± 2.6% increases in biomass production (g dry cell weight/liter), cell density (cells/ml) and growth rate, respectively, in low light with similar improvements in high light (81 ± 5.5, 103 ± 12.4 and 65 ± 3.8% increases in biomass production, cell density and growth rate, respectively) (Supplementary Fig. S6 f–h).

In mass culture, cells at the surface layer exposed to high irradiance are susceptible to photo-oxidative damage, whereas cells deeper inside the culture are light-limited. Both suboptimal conditions can reduce the light conversion efficiency by as much as 95%^1^,^6^. Thus, the balance between the absorption and utilization of light energy is important for improving photosynthetic productivity. We performed mass cultivation of PTS42 using a photobioreactor to analyse the photosynthetic performance of this strain in the high cell density culture. The biomass production in the mass culture of PTS42 under photoautotrophic condition with 5% CO_2_ was improved by 91 ± 14.0% compared to the wild-type strain (CC-125) (Fig. 4d and Supplementary Fig. S7), which is higher than previous approaches using targeted genetic engineering^9^,^20^ or adaptive laboratory evolution^21^. The result indicates that the strains obtained by phototaxis-assisted screening exhibit improved acclimation ability to variable light intensities in high cell density cultures as they showed highly improved photosynthetic performances in both low and high light unlike truncated antenna mutants reported previously^20^,^30^. Interestingly, the PTS23 and PTS42 exhibited increases in both biomass production (28 ± 4.4 and 146 ± 1.0%) and lipid contents (21 ± 1.3 and 227 ± 3.0%) under nitrogen starvation, resulting in increased total lipid production by 55 ± 1.7 and 706 ± 7.4% compared to the wild-type strain (CC-125), respectively (Fig. 4e and Supplementary Fig. S8). This result indicates that high photosynthetic performances of strains isolated by phototaxis-assisted screening can contribute to increasing lipid production under nitrogen starvation as previously reported^31^.

**Figure 5:**
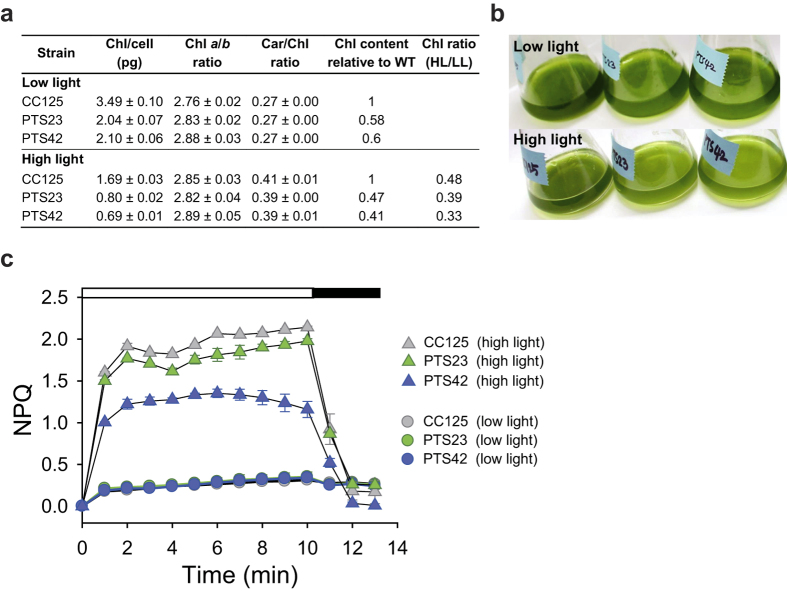
Pigment contents and non-photochemical quenching (NPQ) of the wild-type strain and the two mutants (PTS23 and PTS42) according to different light conditions. Cells were grown photoautotrophically in low (50 μmol photons m^-2 ^s^-1^) and high (300 μmol photons m^-2 ^s^-1^) light. (**a**) Chlorophyll contents (per cell), chlorophyll a/b ratio, chlorophyll/carotenoid ratio contents (**b**) Pictures showing flask cultures of the wild-type strain and the two mutants grown in low (50 μmol photons m^-2 ^s^-1^, upper image) and high (300 μmol photons m^-2 ^s^-1^, lower image) light. All cultures were adjusted to the same cell density (1 × 10^7 ^cells/ml). (**c**) NPQ in the wild-type strain and the two mutants grown in high light (HL, 300 μmol photons m^-2 ^s^-1^) and low light (LL, 50 μmol photons m^-2 ^s^-1^), indicating less heat dissipation of the selected mutants in high light compared to the wild type. The cells were exposed to an actinic light of 800 μmol photons m^-2 ^s^-1^ (white bar) followed by darkness (black bar). Data and errors are mean ± SD of three biological replicates.

The chlorophyll a/b and carotenoid/chlorophyll ratios in the PTS23 and PTS42 were similar to those in the wild-type strain (CC-125) under both light conditions, but chlorophyll contents in mutants were lower than that in the wild-type strain by 40–42% in low light and 53–59% in high light, respectively (Fig. 5a,b). Considering that the wild-type strain exhibits reduced chlorophyll content for acclimation to high irradiance^30^,^32^,^33^, further reduced chlorophyll contents in these mutants reflect more active responses to high light. Taken together, these results suggest that the mutants regulate their photosystem rather than decreasing antenna size^33^. Moreover, the PTS23 and PTS42 exhibited substantially lower non-photochemical quenching (NPQ) than the wild-type strain (CC-125) in high light, whereas no noticeable differences in NPQ were observed between the mutants and the wild-type strain in low light (Fig. 5c). NPQ is a protective mechanism that quenches singlet-excited chlorophylls and harmlessly dissipates excess excitation energy as heat when light energy absorption exceeds the capacity for light utilization in photosynthetic eukaryotes^34^. The absorption of excess light energy results in heat dissipation via NPQ process, but subsaturating light does not induce NPQ^1^,^34^,^35^. Although it is required for photoprotection^34^,^35^, NPQ is regarded as the primary cause of decreases in the photosynthetic efficiency^4^,^6^,^33^. Given their improved growth, this result indicates that the PTS23 and PTS42 can utilize light energy more efficiently for photosynthesis than the wild-type strain, with less heat dissipation in excess light.

We further investigated which genes were altered in the mutants exhibiting the best growth kinetics. In comparison to chemical and uv-induced mutagenesis, insertional mutagenesis has proved to be a very useful tool in forward genetics studies, which aim to identify target genes, because insertional mutagenesis allows the isolation of sequence flanking the mutation with inserted genes as molecular tag by simple PCR-based method^36^. We identified genes that would be responsible for rapid phototactic responses in the 13 of 25 mutants. Classification of genes based on the *Chlamydomonas* genome sequence^37^ and genome annotation (Joint Genome Institute (JGI) *Chlamydomonas* v4.0) provides the candidate processes associated with the improved phototactic responses, including transcriptional regulation, light-signal transduction, flagellar function and membrane transport (Table 1 and Supplementary Fig. S9). We also observed that one additional mutant has the same flanking sequence as the PTS23 mutant, supporting that we can improve selection efficiency by enrichment of mutants showing fast phototactic responses and improved photosynthetic efficiency after phototaxis-assisted screening. Further characterization will reveal whether sensitized phototaxis or improved operating efficiency at the photosynthetic machinery is the primary target in the mutants, thereby providing new strategies for microalgal strain development.

## Discussion

Improving solar energy conversion efficiency of microalgae remains a major challenge to make microalgal biofuel economically attractive. Although intensive efforts have been made to understand algal photosynthetic metabolism, it is still difficult to predict the targets to be manipulated and apply knowledge-based targeted genetic approaches for increasing photosynthetic productivity^4^. Furthermore, engineering of microalgal strain has been limited by the lack of efficient genetic tools such as nuclear transformation and homologous recombination techniques^7^. Thus, most attempts to improve microalgal strains have employed random mutagenesis that requires high-throughput screening methods for selecting strains with substantially enhanced productivity. Currently, high-throughput screening methods depend on characterization of individual strains by measurement of physical or chemical properties using microplates. However, this method is laborious, time-consuming and expensive even in the era of automated high-throughput screening, thus unsuitable for the improvement of strain by screening large mutant libraries. In this context, our screening method provides a powerful strategy for the selection of highly productive algal strains. We designed this method to be simple and easy to use. Therefore, a single researcher could complete overall process of the phototaxis-assisted screening up to 5 cycles within a single day. Due to the increased selection efficiency by enrichment of the candidate strains, mutants with substantially improved photosynthetic yield could be isolated with a high probability within 3 weeks using our method, whereas it normally takes 4–5 months using traditional screening methods. In this study, 10^4^ mutants were grown individually in separate wells of microplates, and the cultures were collected before the screening process to minimize disappearance of mutants by natural selection during mixed culture and ensure that competitive phototaxis-assisted screening is applied to all generated mutants. However, our strategy can be directly applied to the mixed culture of mutants obtained by collecting colonies after transformation, thereby reducing time and cost for screening and improving efficiency of overall operation.

The isolated strains possess highly desired traits for biofuel production because they showed substantial increases in both biomass (up to 2.46-fold) and lipid contents (up to 3.27-fold) under nitrogen starvation, resulting in greatly improved lipid production (up to 8.06-fold) compared to the parental strain. Considering that most genetic engineering approaches to increase lipid biosynthesis have resulted in reduced growth^22^, the traits of the mutants isolated by phototaxis-assisted screening have great potential to improve the economic viability of microalgal biofuels by greatly reducing the overall cost of the biofuel production process. Moreover, we note that our approach can be applied to the development of other useful microalgal strains with phototactic ability such as *Haematococcus pluvialis*, which is well known for its high content of astaxanthin^38^. We believe that this strategy will accelerate microalgal strain improvement by rapid isolation of highly productive microalgal strains and identification of beneficial genetic modifications for further metabolic engineering of microalgal strains, which is essential to enhance microalgal photosynthetic productivity and achieve economically feasible biofuel production from CO_2_.

## Methods

### Strains and culture conditions

*Chlamydomonas reinhardtii* wild-type strain CC-125 was obtained from *Chlamydomonas* Resource Center (University of Minnesota). Cells were grown either photomixotrophically in Tris-acetate-phosphate (TAP) medium^39^ or photoautotrophically in Tris-phosphate (TP, TAP medium without acetate) medium at 23 °C. To synchronize circadian clock, cells were acclimated to LD cycle (12-h light: 12-h dark). Cells were grown in low (50 μmol photons m^-2 ^s^-1^) or high light (300 μmol photons m^-2 ^s^-1^) for physiological measurements. A thin-film photobioreactor (110 cm in height and 15 cm in diameter) equipped with a sparger for gas supply was used to perform photoautotrophic mass culture at a light intensity of 350 μmol photons m^-2 ^s^-1^, measured at the surface of photobioreactor. Cell density was measured using an improved Neubauer hemocytometer or by optical density at 800 nm. Maximum growth rates were calculated by fitting growth curves to the Gompertz function^40^. For the estimation of biomass production, 10 ml of culture was vacuum filtered through a glass-fibre filter (GF/C, Whatman). The filter was then washed twice with deionized water to remove adhering salts, dried in an oven at 70 °C for 48 h and weighed.

### Measurements of chlorophyll fluorescence, O_2_ evolution activity and pigment contents

Chlorophyll fluorescence was measured with an FMS2 fluorometer (Hansatech). Cells grown to exponential phase (30 μg of chlorophyll *a*) were loaded onto a glass-fibre filter, and the filter was placed on the leaf clip. For determination of the PSII operating efficiency (Y(II)), cells (without dark-adaptation) were exposed to stepwise-increasing actinic light (from 1 to 900 μmol photons m^-2 ^s^-1^) for 20 s at each light intensity, and a saturating flash (3,000 μmol photons m^-2 ^s^-1^, 0.7 s duration) was applied to measure Fm’. Y(II) was calculated as (Fm’–Fs)/Fm’. For measurement of NPQ, cells were adapted to dark condition for 30 min. After 5 min of far-red light, cells were exposed to actinic light of 800 μmol photons m^-2 ^s^-1^ for 10 min, followed by darkness for 5 min with a saturating flash every 1 min. NPQ was calculated as (Fm–Fm’)/Fm’. Oxygen evolution was measured with Oxygraph Plus (Hansatech) as described^41^ after addition of NaHCO_3_ (10 mM final concentration). Apparent quantum yield of oxygen evolution (*α*) was determined as the slope of the linear portion of the curve generated by plotting oxygen evolution against light intensity. Maximum photosynthetic rate (P_max_) was determined as net oxygen evolution at 350 μmol photons m^-2 ^s^-1^ plus dark respiration. The cellular chlorophyll and carotenoid contents were determined spectrophotometrically as described^30^,^32^.

### Fabrication of microfluidic device

A monolayer PDMS microfluidic device was fabricated using standard soft photolithography^42^. The design of microstructure was generated using AutoCAD software and printed on transparent photomask film. We fabricated a silicon mould master using SU-8 negative photoresist (SU-8 50, Microchem) on silicon wafers. The PDMS prepolymer (10:1 mixture of 184 Sylgard base and curing agent, Dow Corning) was poured on the SU-8 mould and cured thermally at 80 °C. The PDMS layer containing microchannel was bonded to a glass slide using oxygen plasma. The microfluidic device was composed of an inlet chamber (4 mm in diameter), outlet chamber (4 mm in diameter) and microchannel (30 mm in length, 50 μm in height) including observation zone (channel length 300 μm, width 100 μm, height 50 μm) near the outlet chamber. The width of microchannel gradually decreased from 4 mm (at the inlet) to 100 μm (at the observation zone) to reduce hindrance of phototactic movement by microchannel and facilitate the monitoring of phototactic response at the single-cell resolution level.

### Analysis of phototactic response

Cells were grown exponentially for 3 days photomixotrophically under continuous light at a light intensity of 50 μmol photons m^-2 ^s^-1^. The culture was then diluted to a density of 1.65 × 10^5 ^cells/ml and dark-adapted for 30 min to make cells sensitive to light stimulus. A 40 μl aliquot of the diluted culture was loaded into the inlet chamber of the microfluidic device, and an equal volume of TAP medium was loaded into the outlet chamber. Cells were collected into the inlet chamber via exposure to green LED light at the end of the outlet chamber. After hydrostatic balance was established in the microchannel, a green LED (70 μmol photons m^-2 ^s^-1^) was illuminated at the end of the inlet chamber to evoke negative phototactic responses. The phototactic movements of cells were monitored and recorded under an inverted microscope (Olympus CKX41) equipped with a digital video camera (Canon EOS 700D). Cells arriving at observation zone near the outlet chamber were counted, and their arrival times were automatically recorded for 30 min using custom software. From the histogram of phototactic cells, skewness of arrival time distribution, indicating asymmetry of distribution, was calculated using the skew function (Excel, Microsoft). Inverse average arrival time, indicating how fast cells of each strain respond and move by exposure to the light stimuli, was calculated as (average arrival time)^–1^.

### Mutagenesis

Nuclear transformation was performed by electroporation^43^ for the generation of insertional mutants. The transformation cassette carrying the hygromycin resistance gene, *aph7*″, was PCR amplified from pHyg3^44^ using primers (hyg3F: 5′-CAAGCTTCTTTCTTGCGCTATGA-3′, hyg3R: 5′-AAGCTTCCATGGG ATGACGGG-3′). Cells (CC-125) were grown to early exponential phase (1 × 10^6 ^cells/ml) and treated with autolysin. The cells were then resuspended in TAP medium supplemented with 40 mM sucrose to 1 × 10^8 ^cells/ml. For electroporation, 300 ng of marker gene was added to 40 μl of cells in an electroporation cuvette with 2 mm gap. An electric pulse of 250 V with 8 ms pulse length was applied to the sample using an ECM830 electroporator (BTX Harvard Apparatus). After electroporation, the cells were resuspended in 10 ml of TAP medium with 40 mM sucrose and incubated under dim light for 16 h. Transformants were selected on TAP agar plates containing 15 μg/ml hygromycin B.

### Phototaxis-assisted screening

Each colony on agar plates was transferred into 1 ml of TAP medium containing hygromycin B (2 μg/ml) in each well of 24-well microplate and cultured under low light condition (50 μmol photons m^-2 ^s^-1^) at 23 °C. All cultures of 10,000 transformants were then mixed, centrifuged and resuspended in 6 ml of TAP medium to a cell density of 6.11 × 10^9 ^cells/ml. For screening of the mixture of 10,000 transformants, a microfluidic device with enlarged chambers (8 mm in diameter) and a microchannel (width from 8 mm to 0.4 mm, height 100 μm) was used. A 2.75 ml aliquot (1.68 × 10^10 ^cells) of dark-adapted cell mixture was loaded into the inlet chamber and exposed to green LED light (70 μmol photons m^-2 ^s^-1^) for 10 min to isolate cells showing fast phototactic responses. The cells arrived at the outlet chamber within 10 min were collected by pipetting and recovered in TAP medium for 2 h. The cells were then used for the next cycle of phototaxis-assisted screening. After five cycles of phototaxis-assisted screening, we obtained 3 ml of cell mixture with a cell density of 2.69 × 10^5 ^cells/ml, and a 100 μl aliquot of the culture was plated onto TAP agar containing hygromycin B (15 μg/ml) for the isolation of mutants as separated colonies. To compare the growth of mutant mixtures between each cycle of phototaxis-assisted screening, cells were inoculated to a density of ~1 × 10^4 ^cells/ml and grown photoautotrophically.

### Analysis of genomic DNA

Genomic DNA was extracted using a method described previously^45^. Thermal asymmetric interlaced (TAIL)-PCR^36^,^46^ or inverse PCR^47^ was used to identify flanking sequences. To confirm the integration site, PCR analysis was performed using sequence-specific primers targeting the flanking sequences on the both sides of integrated marker gene (Supplementary Table S1 for all oligonucleotide sequences), and amplified product was sequenced (Genotech). For inverse PCR, 0.5 μg of genomic DNA was digested with SacI in a 100 μl reaction at 37 °C overnight. Following ethanol precipitation, 100 ng of digested DNA was self-ligated with T4 DNA ligase in a 100 μl reaction at 16 °C overnight. For Southern blot analysis, 10 μg of genomic DNA was digested with PstI or NsiI in a 1 ml reaction volume at 37 °C with overnight incubation. The digested DNA was ethanol-precipitated, electrophoresed and then blotted onto a positively charged nylon membrane (Zeta-Probe; Bio-Rad). Probes were obtained by PCR amplification using specific primers (HgPrF: 5′-AGGTCTTCCCGGAACTGCTG-3′, HgPrR: 5′-AGAGGAACTGCGCCAGTTCC-3′). Probe labelling, hybridization, washing and signal detection were performed following manufacturer’s protocol.

### Lipid analysis

Cells were grown photoautotrophically for 4 days in the presence of 5% CO_2_ in TP medium under low light condition (50 μmol photons m^-2 ^s^-1^) at 23 °C. The cells were then harvested, resuspended in TP(-N) (TAP without acetate and nitrogen) medium and incubated for 4 days for lipid accumulation. An 8 ml aliquot of cells was harvested, and total lipids were extracted using the Bligh-Dyer method^48^. FAME (fatty acid methyl esters) was prepared by acid-catalysed transesterification of the total lipids. Two millilitres of methanolic sulphuric acid (3%, v/v) was added to a 15 ml screw-capped glass tube containing the total lipid extract in 1 ml of hexane spiked with 1 mg of pentadecanoic acid as an internal standard. The mixture was vortexed and heated at 95 °C for 1.5 h. After cooling, 2 ml of water and hexane were added, and FAMEs were separated by collecting the organic phase. The extracted FAMEs were analysed using a gas chromatograph (Agilent 7890A) equipped with a flame ionization detector and a Stabilwax column (Restek) with the following conditions: injection volume, 1 μl; split ratio 1:50; injector temp 250 °C; detector temp. 270 °C; oven temperature, hold at 200 °C for 5 min, increase to 230 °C at 20 °C/min and hold at 230 °C for 13 min. FAMEs were identified by retention time and comparison with FAME standards (Sigma).

## Additional Information

**How to cite this article**: Kim, J. Y. H. *et al.* Microfluidic high-throughput selection of microalgal strains with superior photosynthetic productivity using competitive phototaxis. *Sci. Rep.* **6**, 21155; doi: 10.1038/srep21155 (2016).

## Supplementary Material

Supplementary Information

Supplementary Video S1

Supplementary Video S2

Supplementary Video S3

Supplementary Video S4

## Figures and Tables

**Table 1:** Genomic DNA analysis of integration sites in 13 selected PTS (phototaxis-screening) mutants.

Mutant	Location	Protein ID^b^	Function^c^	Methods for sequence determination
PTS1	promoter (B)^a^	516748	Signal recognition particle receptor	TAIL-PCR & PCR-SP^d^
PTS23	promoter (B)	525919	Similar to homeobox A3 (*Mus musculus*)	Inverse PCR & PCR-SP
PTS26	17^th^ intron (B)	516786	Ionotropic glutamate receptor	TAIL-PCR & PCR-SP
PTS36	5′-UTR (B)	519251	Putative glycine-rich cell wall structural protein	TAIL-PCR & PCR-SP
PTS37	5′-UTR (B)	519327	Glycoside hydrolase	TAIL-PCR & PCR-SP
PTS42	promoter (B)	516641	Protein binding; PHD-type zinc finger	TAIL-PCR & PCR-SP
PTS59	3^rd^ intron (S)	523869	Diacylglycerol acyltransferase family	TAIL-PCR
PTS61	3′-UTR (B)	513005	Vesicle-mediated transport; t-SNARE family	TAIL-PCR & PCR-SP
PTS64	2^nd^ intron (B)	520695	Biopterin transport-related protein BT1	TAIL-PCR & PCR-SP
PTS66	3′-UTR (B)	518826	Flagellar associated protein	TAIL-PCR & PCR-SP
PTS69	5′-UTR (S)	515661	3-phosphoshikimate 1-carboxyvinyltransferase	TAIL-PCR
PTS118	5′-UTR (B)	513996	Nucleosome assembly; Histone H4	TAIL-PCR & PCR-SP
PTS124	6^th^ exon (B)	512634	GDP mannose transporter	TAIL-PCR & PCR-SP

^a^B: both flanking sequences were identified, S: single flanking sequence was identified.

^b^Protein IDs were obtained from JGI *Chlamydomonas* v4.0 (encoded by gene closest to the insertion site).

^c^Function (domain, motif) was described based on the JGI *Chlamydomonas* v4.0.

^d^PCR-SP: PCR using specific primers (Supplementary Table S1).
