# Supplementary material for: Microfluidic high-throughput selection of microalgal strains with superior photosynthetic productivity using competitive phototaxis
Source: Sci Rep. 2016 Feb 8;6:21155. doi: 10.1038/srep21155 (PMC4745075; doi:10.1038/srep21155)
Supplement: Supplementary Information [file srep21155-s1.pdf]

# Supplementary Information

# Microfluidic high-throughput selection of microalgal strains with superior photosynthetic productivity using competitive phototaxis

**Jaoon Young Hwan Kim[1,†], Ho Seok Kwak[1,†], Young Joon Sung[1], Hong Il Choi[1], Min Eui Hong[1], Hyun Seok Lim[1], Jae-Hyeok Lee[2], Sang Yup Lee[3], and Sang Jun Sim[1,4,*]**

[1]Department of Chemical and Biological Engineering, Korea University, Seoul, 136-713, Republic of Korea

[2]Department of Botany, University of British Columbia, Vancouver, V6T1Z4, Canada

[3]Department of Chemical and Biomolecular Engineering (BK21 Plus Program), BioProcess Engineering Research Center, Bioinformatics Research Center, Center for Systems and Synthetic Biotechnology, Institute for the BioCentury, Korea Advanced Institute of Science and Technology, Daejeon 305-701, Republic of Korea

[4]Green School, Korea University, Seoul, 136-713, Republic of Korea

*Corresponding author: simsj@korea.ac.kr

[†]These authors contributed equally to this work.

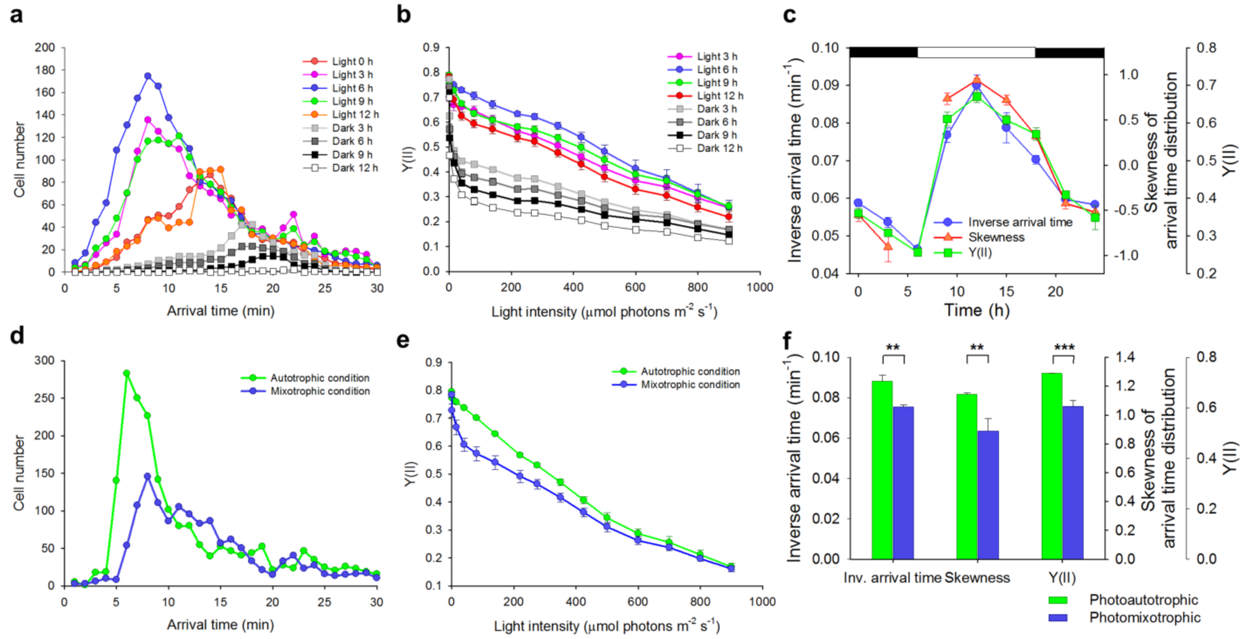


**Supplementary Figure S1. Effect of circadian rhythm and trophic conditions on phototactic response and photosynthetic activity.** (**a**) Distribution of the number of phototactic cells according to arrival time under LD cycle (12 h–12 h). Cells grown photomixotrophically in low light (50 μmol photons $m^{-2}$ $s^{-1}$) were harvested every 3 h. The phototactic response was measured on 6,600 cells per analysis using microfluidic device. (**b**) PSII operating efficiency (Y(II)) measured under LD cycle (x-axis: stepwise-increases in actinic light from 1 to 900 μmol photons $m^{-2}$ $s^{-1}$). (**c**) Skewness of arrival time distribution, inverse average arrival time of phototactic cells (wild type, CC125) and PSII operating efficiency (Y(II)) plotted against culture time under LD cycle (12 h light (white bar)–12 h dark (black bar)). Skewness of arrival time distribution at the end of dark phase (6 h) was not determined due to the near absence of a response. (**d**) Distribution of phototactic cell number according to arrival time under photoautotrophic (green) and photomixotrophic (blue) conditions. The phototactic response was measured on 6,600 cells per analysis. (**e**) PSII operating efficiency (Y(II)) measured under photoautotrophic (green) and photomixotrophic (blue) conditions (x-axis: stepwise-increases in actinic light from 1 to 900 μmol photons $m^{-2}$ $s^{-1}$). (**f**) Skewness of arrival time distribution, inverse average arrival time of phototactic cells (wild type) and Y(II) under photoautotrophic conditions (green bars) compared to those under photomixotrophic conditions (blue bars). ** $P<0.01$; *** $P<0.001$, two-tailed Student's t-test. Cells were grown under continuous low light condition (50 μmol photons $m^{-2}$ $s^{-1}$) (**d–f**). All data and error bars are the mean ± SD of three biological replicates.

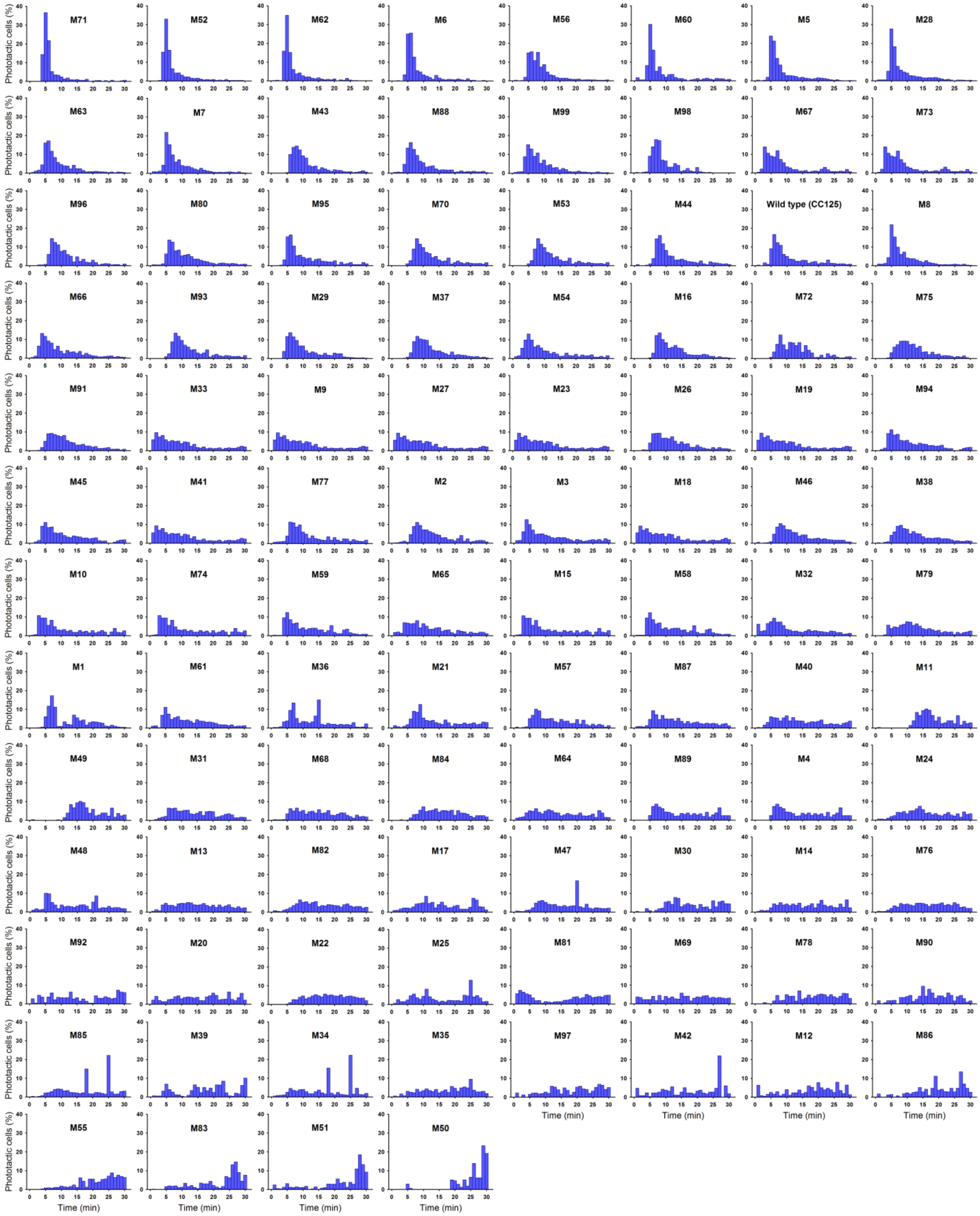


**Supplementary Figure S2. Histograms showing the phototactic responses of 100 strains with different photosynthetic activities.** The negative phototactic responses of 100 strains,

including the wild-type strain and 99 randomly selected mutants with a wide range of PSII operating efficiency (Y(II)), were analyzed, and histograms of phototactic cells (% of total phototactic cells) as a function of their arrival time are shown. Cells were grown photomixotrophically under continuous low light condition (50 µmol photons $m^{-2}$ $s^{-1}$). The phototactic response was measured on 6,600 cells per analysis. All data are the mean of three biological replicates.

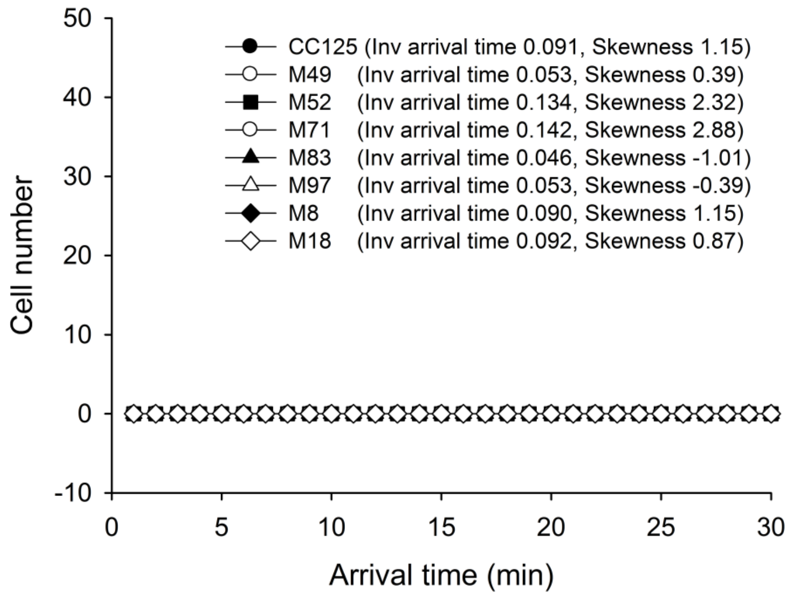


**Supplementary Figure S3. Responses of various strains in microfluidic device without exposure to light stimulus.** The responses of the wild-type strain and mutants with different phototactic responses were monitored without a light stimulus. Cells were grown photomixotrophically under low light condition (50 µmol photons $m^{-2}$ $s^{-1}$). The response was measured on 6,600 cells per analysis. All data and error bars are mean ± SD of three biological replicates.

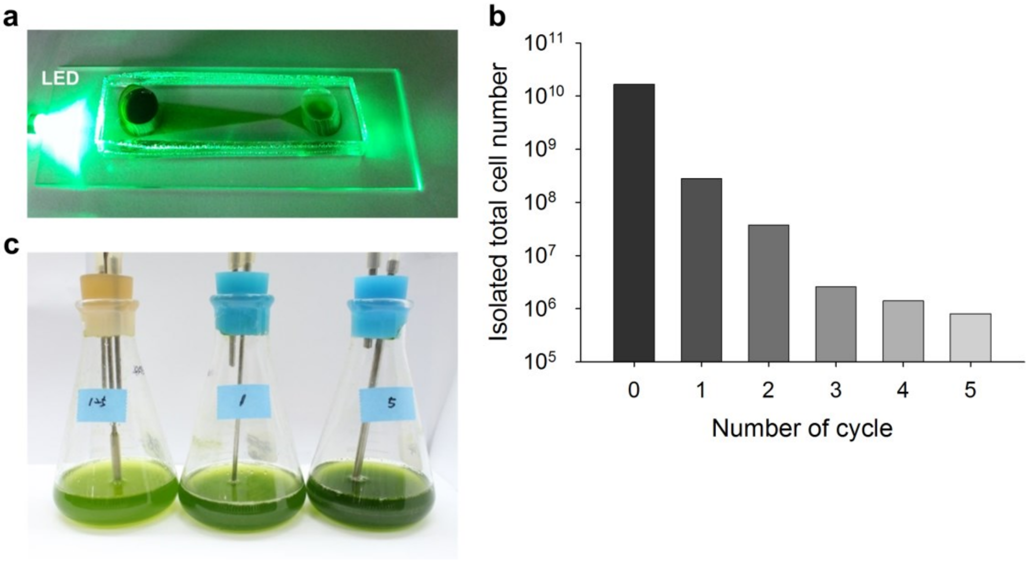


**Supplementary Figure S4. Phototaxis-assisted screening.** (**a**) Picture showing a microfluidic device with a green LED used for phototaxis-assisted screening. After dark-adaptation for 30 min, mutant mixture was loaded into the left chamber (dark green) and exposed to green LED light (70 µmol photons $m^{-2}$ $s^{-1}$) for 10 min to isolate strains showing fast phototactic responses at the right chamber (light green). (**b**) Total number of cells isolated from mutant mixture after each cycle of screening. (**c**) Picture showing flask cultures of the wild-type strain and mutant mixtures obtained after 1 and 5 cycles of screening. The wild-type strain and mutant mixtures with the same initial cell densities (~$1 \times 10^4$ cells/ml) were grown photoautotrophically under continuous low light condition (50 µmol photons $m^{-2}$ $s^{-1}$).

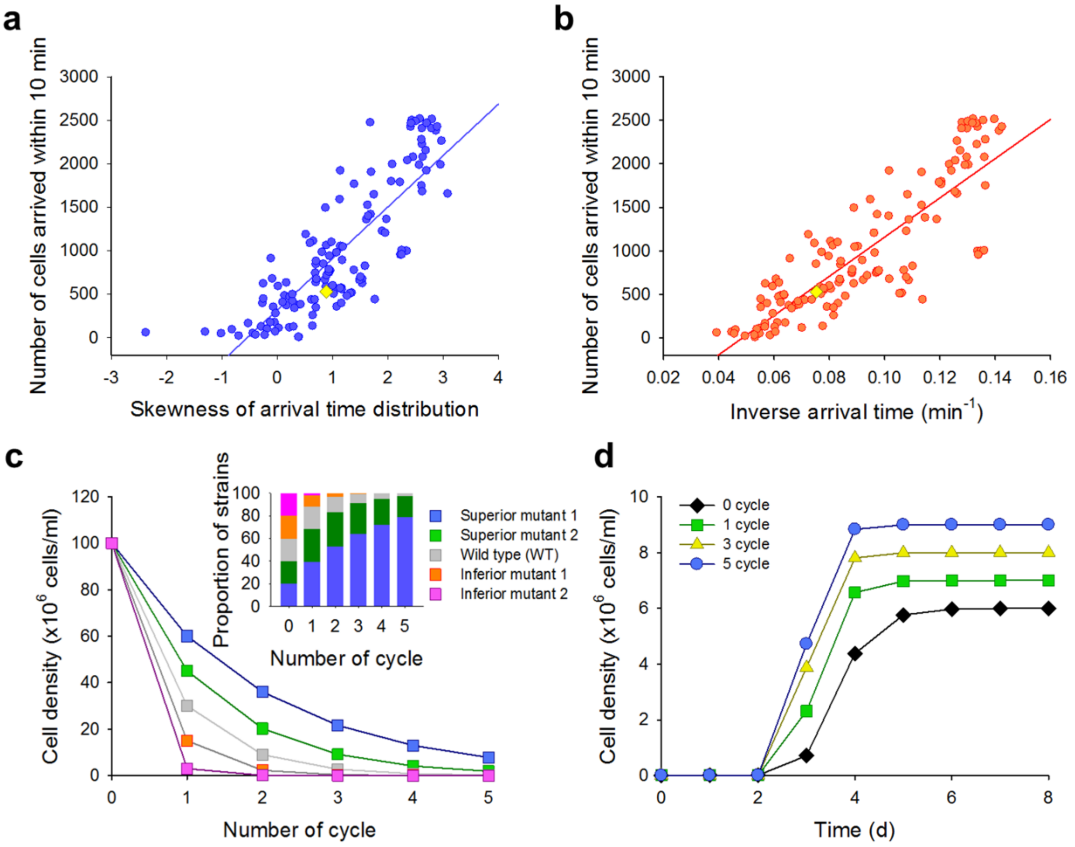


**Supplementary Figure S5. A fast phototactic response provides a competitive advantage by increasing population and fitness.** (**a**) Correlation between phototactic cell number of 136 strains arrived within 10 min and skewness of arrival time distribution ($R^2$ = 0.70). (**b**) Correlation between phototactic cell number of 136 strains arrived within 10 min and inverse average arrival time ($R^2$ = 0.73). The phototactic response was measured on 6,600 cells per analysis, and all data are the mean of three biological replicates. 136 strains include the wild-type strain (yellow diamond), 99 randomly selected mutant without phototaxis-assisted screening, and 36 strains isolated after five cycles of phototaxis-assisted screening (**a**,**b**). Cells were grown photomixotrophically under continuous low light condition (50 µmol photons $m^{-2}$ $s^{-1}$). (**c**) Simplified model showing the changes in the cell densities of five strains with different phototactic responses in the phototaxis-assisted screening ($C_n = C_0(R_a \times R_p)^n$, where $C_n$ is the cell density of each strain after n cycles of phototaxis-assisted screening; $C_0$ is the initial cell density of each strain before phototaxis-assisted screening (set to $1 \times 10^8$); $R_a$ is the ratio of cell number arrived within 10 min to total cell number in the wild type (set to 0.3); $R_p$ is the ratio between cell number arrived within 10 min in each mutant and the wild type (set to 2, 1, 0.5 and 0.1 for 4 mutants). The inset shows the population dynamics of each strain in the mixture of these strains according to number of screening cycle. The proportion of each mutant in the population was obtained from (**c**). Because the cell number arrived within 10 min depends on the rate of phototaxis (**a**,**b**), the population of strains showing fast responses increases as the number of screening cycle increases (**c** inset). (**d**) Simplified model showing growth curves for mixtures of

five strains after different number of screening cycle. The cell density of each mutant mixture after n cycles of screening was calculated using a modified Gompertz function[40], $C_n = C_0 \exp[A \cdot \exp\{-\exp(\mu_{max} \cdot e \cdot (\lambda - t)/A + 1)\}]$. The model assumes that the $\mu_{max}$ of strain mixture is average $\mu_{max}$ of strains (set to 2, 1.5, 1, 0.5 and 0.1), weighted by the proportion of each strain in the population after each cycle of phototaxis-assisted screening (**c**). A is the ratio of the final cell density of strain to the initial cell density ($\ln(C_f/C_0)$) after each cycle, which has the same meaning as the fitness of strain (set to 6.4, 6.55, 6.68 and 6.8 for 0, 1, 3 and 5 cycle based on the relatively high fitness of strains exhibiting fast phototactic responses).

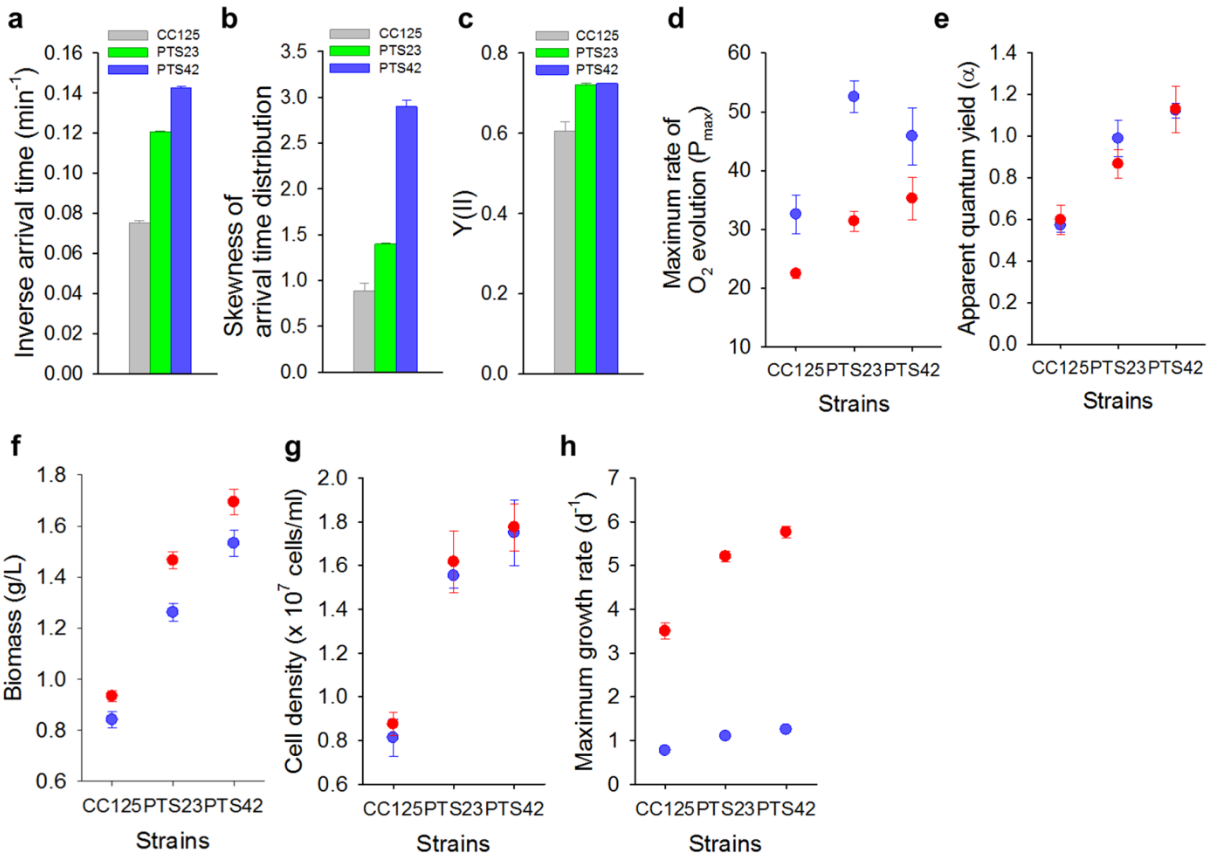


**Supplementary Figure S6. Phototactic and photosynthetic characteristics of two mutants (PTS23, PTS42) compared to the wild-type strain.** (**a–c**) Inverse average arrival time (**a**), skewness of arrival time distribution (**b**) and PSII operating efficiency (Y(II)) (**c**) of two mutants compared to the wild-type strain. Cells were grown photomixotrophically in TAP medium under continuous low light condition (50 μmol photons $m^{-2}$ $s^{-1}$). (**d**,**e**) Maximum photosynthetic rates ($P_{max}$) (**d**) and apparent quantum yield of oxygen evolution ($\alpha$) (**e**) on a per-cell basis in the two mutants compared to the wild-type strain under continuous low light (50 μmol photons $m^{-2}$ $s^{-1}$) and high light conditions (300 μmol photons $m^{-2}$ $s^{-1}$). $\alpha$, arbitrary unit; $P_{max}$, nmol $O_2$ ($10^6$ cells)$^{-1}$ $min^{-1}$. (**f–h**) Biomass production (**f**), final cell density (**g**), maximum growth rate (**h**) of the two mutants compared to the wild-type strain in continuous low and high light. Maximum growth rates were calculated from the Gompertz function[40] (**h**). Cells with the same initial cell densities (~5 × $10^5$ cells/ml) were grown photoautotrophically in a $CO_2$ incubator at 5% $CO_2$. Blue circle: low light, Red circle: high light (**d–h**). All data and error bars are the mean ± SD of three biological replicates.

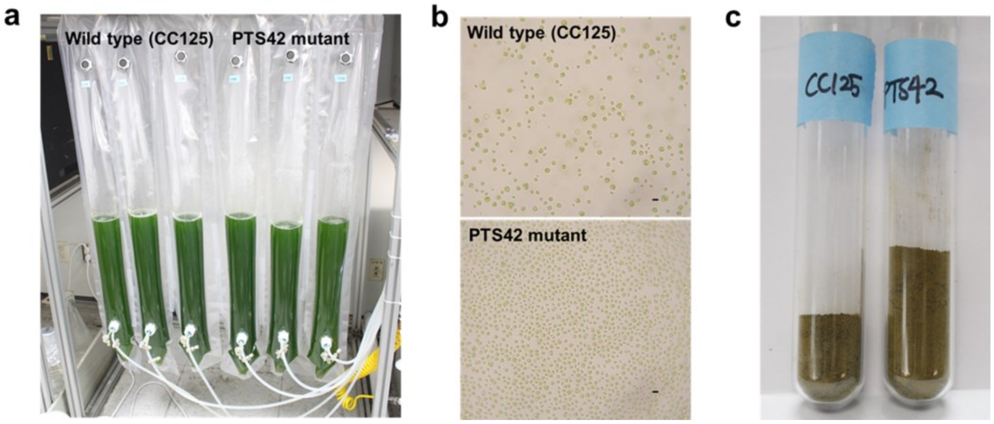


**Supplementary Figure S7. Mass culture of the wild-type strain and PTS42 mutant using photobioreactor system.** (**a**) Picture showing cultivation of wild type and PTS42 in photobioreactors. (**b**) Representative microscopy images showing different cell densities of wild type and PTS42 mutant at 72 h. Scale bars, 10 µm. (**c**) Picture showing dried cell powder obtained from 2-liter culture broth of wild type and PTS42 mutant. Cells were grown photoautotrophically with the same initial cell densities (~$1 \times 10^5$ cells/ml) in 3-liter TP medium using a 5-liter photobioreactor at a light intensity of 350 µmol photons $m^{-2}$ $s^{-1}$ by supplying 5% $CO_2$-enriched air at a flow rate of 50 ml $l^{-1}$ $min^{-1}$.

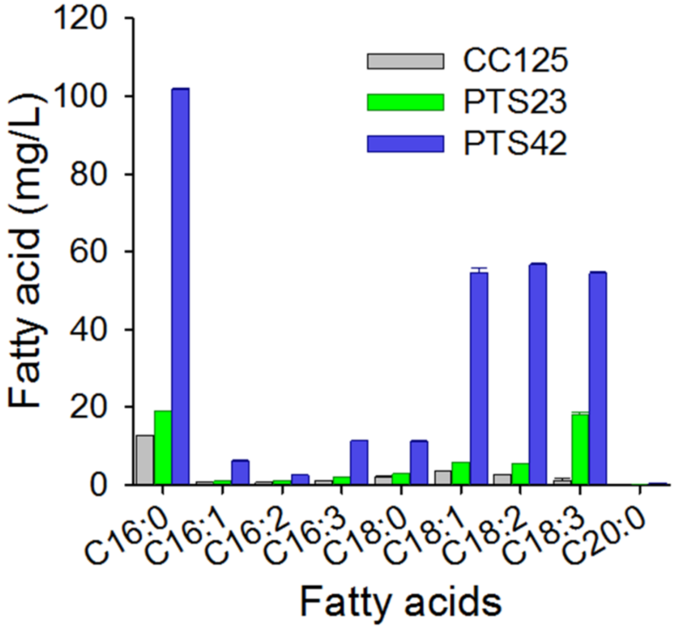


**Supplementary Figure S8. Fatty acid production in two mutants (PTS23, PTS42) compared to the wild-type strain.** Cells were grown photoautotrophically for 4 days in a $CO_2$ incubator at 5% $CO_2$ in TP medium under continuous low light condition (50 µmol photons $m^{-2}$ $s^{-1}$), and then incubated in TP(-N) media in a $CO_2$ incubator at 5% $CO_2$ for 4 days for lipid accumulation. All data and error bars are mean ± SD of three biological replicates.

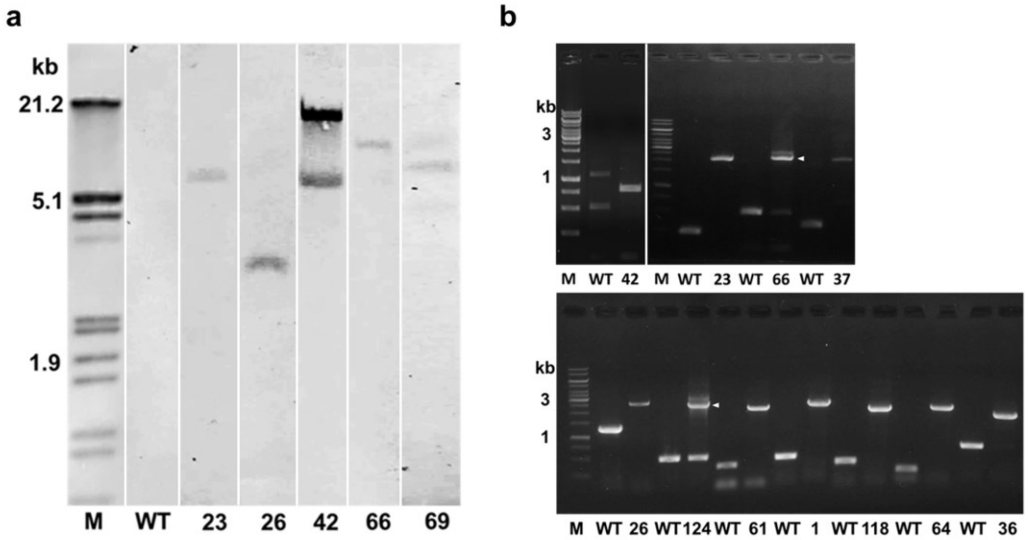


**Supplementary Figure S9. Genomic DNA analysis of selected PTS (phototaxis-screening) mutants.** (**a**) Southern blot analysis of genomic DNA from five selected PTS mutants (PTS23, 26, 42, 66, 69). Genomic DNA was digested with PstI (PTS23, 42, 66) or PstI & NsiI (PTS26, 69). The 3′ region of the *aph7″* coding sequence was used as a probe. M: Dig-labelled DNA molecular weight marker III (Roche), WT: wild type (CC125). Each number denotes the number of PTS mutants. (**b**) PCR analysis of insertion site in the genomic DNA of PTS mutants. PCR was carried out using specific primers (Supplementary Table S1) targeting flanking sequences on both sides of the marker gene, and amplified product was sequenced. M: GeneRuler 1 kb DNA ladder (Fermentas), WT: wild type (CC125). Each number denotes the number of PTS mutants. 42: PCR product using primers (42R, UP2). The arrow indicates specific amplified product of the mutant. Due to the concatameric insertion of selection marker gene, one additional integration site in PTS42 and one of both flanking sequences in PTS69 were not identified.

## Supplementary Table S1. Sequences of oligonucleotides used in this study.

| Name | Sequence (5'→3') | Description |
|---|---|---|
| UP3 | GACTCACCTCCCAGAATTCCTGG | TAIL-PCR (primary), specific primer for upstream sequence[46] |
| UP2 | TCGTTCCGCAGGCTCGCGTAGG | TAIL-PCR (secondary), specific primer for upstream sequence[46] |
| UP1 | TCGAGAAGTAACAGGGATTCTTGTGTCATG | TAIL-PCR (tertiary), specific primer for upstream sequence[46] |
| DP4 | CTTCGAGGTGTTCGAGGAGACCC | TAIL-PCR (primary), specific primer for downstream sequence[46] |
| DP3 | CGCTGGATCTCTCCGGCTTCACC | TAIL-PCR (secondary), specific primer for downstream sequence[46] |
| DN1 | GAACTGGCGCAGTTCCTCTG | TAIL-PCR (tertiary), specific primer for downstream sequence (this study) |
| RMD227 | NTCGWGWTSCNAGC | TAIL-PCR, degenerate primer[36] |
| iHSU1 | ATGACACAAGAATCCCTGTTACTT | Inverse PCR, for upstream sequence |
| iHSU2 | CATAGCGCAAGAAAGAAGCTTG | Inverse PCR, for upstream sequence |
| iHSD1 | CAGTGCTCGCCGAACAGCTTGA | Inverse PCR, for downstream sequence |
| iHSD2 | CGCTGGATCTCTCCGGCTTCACC | Inverse PCR, for downstream sequence (same as DP3) |
| 1F | ACGCATATTTGTCTTGTGCACACA | Sequence specific primer for mutant PTS1 |
| 1R | AGGTTCGTAGGTCAGGCAAACAGA | Sequence specific primer for mutant PTS1 |
| 23F | AGCCCAGTCACTGTGGAGTCACTTA | Sequence specific primer for mutant PTS23 |
| 23R | GCCCTAGGCAGAGTCCAAAGCT | Sequence specific primer for mutant PTS23 |
| 26F | TGACTCGATCGCTAAATGCGTTG | Sequence specific primer for mutant PTS26 |
| 26R | CCAGCAGAGGTAGGATCCCATTTC | Sequence specific primer for mutant PTS26 |
| 36F | AAACCTAGCTATGGTATCATTTCC | Sequence specific primer for mutant PTS36 |
| 36R | ACTGGCGTCCCTGCAATGAAAGA | Sequence specific primer for mutant PTS36 |
| 37F | GCCCTGCTGTCTTCTGATCTAAGC | Sequence specific primer for mutant PTS37 |
| 37R | GCACGAATACTCACGAGTGAATG | Sequence specific primer for mutant PTS37 |
| 42R | GGACACCAAGATAGCAAGAAGAAGC | Sequence specific primer for mutant PTS42 |
| 61F | TCGAAGAACTGGCAATTCATATGA | Sequence specific primer for mutant PTS61 |
| 61R | CTTGAATCGATTTTCTCTTTGTCAG | Sequence specific primer for mutant PTS61 |
| 64F | ATGCTTGGTCAGACGGATAACGTA | Sequence specific primer for mutant PTS64 |
| 64R | AGTGAGTGACTAGCGGTTGTTTAA | Sequence specific primer for mutant PTS64 |
| 66F | GCCAATCATGCCTGCTGTGAGACG | Sequence specific primer for mutant PTS66 |
| 66R | AGGCCATTACCTTCACTACAGCG | Sequence specific primer for mutant PTS66 |
| 118F | GAGTGAGTATCGCCAAGCAATTGC | Sequence specific primer for mutant PTS118 |
| 118R | CTTCTGTCATGTTGAACCTCTC | Sequence specific primer for mutant PTS118 |
| 124F | TGTTGGGGTGTAGTTGTAGTTGG | Sequence specific primer for mutant PTS124 |
| 124R | CCATGCTGAACTCGTCCATCTGC | Sequence specific primer for mutant PTS124 |

**Supplementary Video S1. Immediate phototactic response of a single cell of *Chlamydomonas reinhardtii* in a microchannel to the changes in the direction of light.**

The movie shows the negative phototactic responses of wild type cells (CC125) at the level of single-cell resolution. The direction of light is changed using two green LED lamps at both ends of the microchannel, which are switched on and off alternately.

**Supplementary Video S2. Analysis of negative phototactic response using custom software.**

The movie shows that cells arrived at observation zone near the outlet chamber are counted, and their arrival times are automatically recorded using custom software.

**Supplementary Video S3. Phototactic responses under a 12 h–12 h LD cycle.**

The movie shows the phototactic responses of wild type cells (CC125) at different time points (see the text in each panel) under the LD cycle. All video panels were recorded during the same period.

**Supplementary Video S4. Comparison of the phototactic responses of two mutants to wild type.**

The movie shows the phototactic responses of two mutants with different phototactic responses (left: slow response, right: fast response) and photosynthetic efficiencies (left: low Y(II), right: high Y(II)) compared to the wild type strain (middle, CC125). All video panels were recorded during the same period.
